# Committed to Health: Key Factors to Improve Users’ Online Engagement through Facebook

**DOI:** 10.3390/ijerph17061814

**Published:** 2020-03-11

**Authors:** Juana Alonso-Cañadas, Federico Galán-Valdivieso, Laura Saraite-Sariene, Carmen Caba-Pérez

**Affiliations:** Mediterranean Research Center of Economy and Sustainable Development (CIMEDES), Department of Economics and Business, University of Almeria, Crta Sacramento s/n, La Cañada de San Urbano, 04120 Almería, Spain; fgalan@ual.es (F.G.-V.); ls703@ual.es (L.S.-S.); ccaba@ual.es (C.C.-P.)

**Keywords:** health communication, health organization, management, social media, Facebook, engagement

## Abstract

Health organizations, continuously exposed to public scrutiny due to the social relevance of the services provided, have adopted social media to disseminate information about health but also about themselves, and thus, reducing uncertainty and improving communication. In this context, users’ participation in social media has become one of the main indicators of their effectiveness, highlighting the importance of analyzing which factors enhance online engagement. This research extends the number of variables identified in prior studies and analyzes 19,817 Facebook posts from 126 health organizations. Using multivariate linear regression, explanatory results show that economic and organizational attributes, and factors related to the social media posts, both contribute significantly to explain the engagement reached in social media by those organizations. According to our findings, health organizations are not taking enough advantage of social media to engage with their current and potential users. The dissemination of relevant information using visually attractive formats could help draw the attention of consumers, both to reach a higher commitment with the organization and to create value for society.

## 1. Introduction

Health organizations (hereinafter, HOs) have witnessed the consolidation of social media as a tool for institutional communication [1]. There is no doubt that health communication is especially sensitive due to its importance and implications, so HOs must respond to the needs of citizens, which demand more participation and information in health [2]. In fact, Health 2.0 is grounded on the idea that medical care must move away from hospital-based attention and focus on promoting health, providing domestic health care and instructing consumers and patients to take responsibility for their own health [3]. All this has generated a new paradigm in health communication in which the HOs, continually exposed to public scrutiny due to the social importance of their services [4], have found in social media a vehicle through which to involve society in the organization.

Social media have become a popular tool for health communication [5], contributing considerably to the value created by HOs and reshaping health information management in a variety of ways, ranging from providing cost-effective ways to improve organization-patient communication and exchange health-related information and experience to enabling the discovery of new medical knowledge and information [6]. From the user’s standpoint, social media are not only a new way of accessing and sharing information [7], but they also encourage the collaboration and participation of stakeholders, increase the connectivity of individuals and allow the direct participation of users in the HOs [8]. In addition, and from an organizational perspective, social media is a valid instrument to: (1) reach and interact with stakeholders, (2) aggregate relevant information from many sources, (3) leverage collaboration, (4) strengthen HOs’ efforts in the field of sustainability [9], promote corporate social responsibility [10] and in delivering important messages to their audience [11].

Among all social media, Facebook (FB) is one of the most used platforms in the health industry, not only because of the large number of users [12], but for being considered a valid tool for the dissemination of messages about health [13,14], including reaching minority groups that otherwise would be very difficult to involve in public health issues [15,16]. In addition, its use for disease surveillance is notorious, encouraging and broadening the field of health studies, being especially used by researchers to address specific health concerns [11].

The inclusion of HOs’ main actors (i.e., patients, professionals and organizations) into their communication strategy via social networks [17] has provided researchers a novel research path. First, previous literature has dealt with the motivations, uses, advantages and disadvantages of this communication channel for health communication [8,18,19,20]. Given the consensus reached on the potential value of social networks [21], a new line of research focused on online engagement has emerged. Social media engagement can be defined as a multi-way interaction between and among an organization and digital communities. Using social media channels facilitates interaction, and health messaging is shared in a way that creates opportunities for information to be acted on by the audience [21]. Its analysis and understanding could lead to better practices that could enhance interactive behaviors, encouraging user engagement with the organization and thus improving the effectiveness of health information dissemination [14]. In addition, it opens a dialogue between society and the organization, which allows both parties to work collaboratively to address issues that affect health and well-being in general. The importance of this concept in the health sector leads to it being considered a tool at the service of the “common good” [22].

In this context, the importance of stimulating interactive behaviors that promote user engagement with the organization through social media, specifically Facebook, is framed. Previous literature has emphasized technical aspects related to the type of publication, the publication schedule, the content disclosed, and other factors related to sociodemographic characteristics, Internet penetration rates or education level, which could influence the user’s online participation through Facebook [23,24].

This set of studies, as shown by [25], has mainly focused on the country of origin of the largest social media, such as the United States and China, as well as developed countries from Europe and the Oceania region. Developed countries represent an interesting environment to analyze online practices regarding health communication since they are generally characterized by having a quality health system and are more likely to have a population in good health. Thus, their health communication management could serve as a reference for other less developed health systems.

Authors such as [1] recognize the progress achieved in relation to the adoption and use of social media by health systems in developed countries. The case of Spain is of particular interest. Its health system is ranked seventh out of 190 countries (according to the ranking of the World Health Organization for 2019), and it is considered an essential pillar of social welfare in Spanish society. The good reputation enjoyed by the Spanish healthcare system at an international level makes it an interesting reference to analyze, particularly regarding how Spanish HOs promote new forms of social communication by using innovative channels to reach a bigger and more engaged audience.

This research analyzes which factors, related to the structural characteristics of HOs and their online communication policy, exert an influence on their users’ online commitment, contributing to expand the literature on social networks and health communication. Furthermore, this study aims to help HOs improve decision-making in communication strategies via social media, in order to achieve a greater social commitment. Following the introduction, the rest of the article is organized as follows: Section 2 encompasses the theoretical background and the development of hypotheses, Section 3 details the research design, in Section 4 results are presented and discussed, and Section 5 details the conclusions and final remarks.

## 2. Literature Review and Hypotheses Development

The literature shows that social media have tremendous potential value in the field of health, primarily because of their ability to allow new forms of access and information exchange, social support and also enhance the collaboration and participation of the stakeholders [8]. Recent research has explored the motivations that lead HOs to integrate social media into their communication strategy, as well as the use of social media by patients, on one side, and health professionals and health organizations on the other [8,26].

In this sense, patients use social networks based on different motivations which can be summarized in the following categories: emotional, information, esteem, network support, social comparison and emotional expression [9]. In general, social media have the potential to empower people to develop healthy lifestyles, make better and more informed medical decisions and improve personal health management [6]. In addition, the use of social networks by patients affects their relationship with healthcare professionals, leading to more equitable communication, thus allowing harmonious relationships and greater interaction [9].

Social networks have allowed HOs to adopt strategies to improve their market share, in addition to contributing to the achievement of their organizational mission and the development of medical care [27]. Previous literature has made it clear that HOs primarily use social media platforms to disseminate health information, advice and suggestions, thus promoting health care, as well as different services and products [23]. Moreover, social networks are also useful for disseminating organizational efforts on sustainability [9] or corporate social responsibility [10].

Despite the benefits derived from the use of social networks in health communication, there are some drawbacks, such as the possible loss of privacy or security in the shared information [28] and the lack of specialized training in both health issues and management of social networks by those involved in managing social media communication [29]. Both HOs and users could lose control of the information disclosed in social media and harmful or inappropriate material may be published in the social network, an event that could affect their reputation and that is difficult to rectify [1].

Be that as it may, social media enhance the interaction between supply and demand in the health sector, and could exert a positive effect, for which HOs must strengthen and properly manage user participation in social networks. Previous studies have analyzed the effect that different technical aspects have on the online participation of users, such as the type of publication, the publication schedule, the content disclosed, as well as other factors related to sociodemographic characteristics, internet penetration rates or level of education. For instance, [24] analyze the features of Facebook posts on Australian public health organizations’ Facebook pages, [14] by performing an analysis about how to improve the level of engagement with US Federal health agencies on Facebook, [23] study the types of content US hospitals post on their Facebook pages and, more recently, [25] explore the factors that facilitate health information diffusion in South East Asia, especially Malaysia.

In summary, and to the best of our knowledge, traditionally, the studies carried out in the context of health communication through social media (basically Facebook) have been descriptive in nature, showing the motivations for their implementation in the health sector, their use, advantages and disadvantages. Under the same descriptive approach, the literature includes aspects related to the type of formats used to publish the information, the frequency of emission according to the days of the week and the publication schedule, as well as the type of content that the HOs disclose. The importance of these studies is notable, since they define the management model that HOs are currently carrying out in their online communication strategy. However, a new set of explanatory studies related to users’ commitment have emerged in the field of health communication. In particular, there is growing interest community research community in understanding which factors (economic and organizational attributes, type of content published in social media, format of posts, among others) explain the user’s commitment to the organization and, therefore, which aspects HOs should emphasize in order to maximize the user’s online participation.

Finally, it should be highlighted that previous literature frame the research mainly from the perspective of several theories: (1) Dialogic Communication Theory [30], which explains how these organizations use social media as a channel to achieve user commitment; (2) Resource Dependence Theory [31], which helps to understand why HOs carry out strategies to improve their relationships with stakeholders, thus reducing uncertainty and resource dependence; (3) Media Richness Theory [32] seeks to explain what type of format should be used by HOs in order to communicate effectively and improve commitment; and, (4) Uses and Gratifications Theory [33] which aims to explain how individuals select particular types of social media and content in order to satisfy their specific needs or desires.

Given this conceptual framework, it is important for HOs to maintain an ongoing dialogue with their stakeholders, working collaboratively to improve the online management of health communication and, thus, redounding in an improvement of the well-being of society [21]. Therefore, it is important to continue studying which factors influence the stakeholder engagement with the organization through social networks. To this end, a set of organizational and social media variables are proposed to be analyzed in order to expand this knowledge (Table 1).

### 2.1. Ownership

Regarding their final objective, public HOs manage their communications differently than their private counterparts [1]. However, public HOs admit that they can also make use of social networks, such as Facebook, to attract their target market [34].

For [24] it is important to examine which strategies are the most effective in achieving user engagement with these types of organizations since, as also concluded by [1], private hospitals achieve better metrics in social networks than public hospitals. This could mean that private hospital marketing campaigns, and the greater investment aimed at generating a more attractive multimedia content, both have a positive impact on users.

Based on these approaches, the following hypothesis is proposed:

**Hypothesis** **1** **(H1).** *HOs’ ownership affects the level of users’ online commitment to HOs via social media.*

### 2.2. Economic Capacity 

According to [35], the relationship between the size of a country´s gross domestic product (GDP) and the density of the installed technology is directly proportional. Consequently, there is a strong relationship between the economic level of the population and its access to internet and new technologies [36]. Furthermore, studies suggest that people living in a more affluent city are more likely to use social networks, with the city’s online activity determined by factors such as individual wealth [37].

However, the increase in the cost of health services seems to be one of the main reasons why people with fewer resources turn to websites, online platforms and other online health-related resources [38]. In addition, both public and private organizations are taking advantage of Medicine 2.0 technological advances to offer health services, due to the significant cost reduction and the resulting impact on national health spending [39].

Therefore, the following hypothesis is posed:

**Hypothesis** **2** **(H2).** *The economic capacity of the population affects the level of users’ online commitment to HOs via social media.*

### 2.3. Size of the Organization

In general, large organizations present a greater ability for survival and growth, due to their better access to the resources needed. In this sense, [40] argue that larger organizations have greater ability to incorporate technology into their communication channel and to use it efficiently to build close relationships with their main stakeholders. These results are supported by [41], concluding that larger organizations attain a higher stakeholder commitment.

Despite this, [42] state that smaller organizations are most likely to adopt social networks and are more interested in implementing social media as a one or two-way communication strategy [43]. Similarly, [44] point out that organizations with a limited budget have found in social media an affordable way to communicate with their members, since one of the motivations for Internet use is to improve the quality of health care but also for cost reduction [8]. In addition, smaller organizations are an example of how social media can be used strategically to involve and engage stakeholders with their goals [45].

Nevertheless, while the only cost in the use of social media is the time involved (in contrast with traditional marketing and communication methods, which also involve costly printing and mailing), larger HOs are more likely to implement official social media accounts [46].

Thus, the following hypothesis states:

**Hypothesis** **3** **(H3).** *The size of the organization affects the level of users’ online commitment to HOs via social media.*

### 2.4. Online Community Size

Organizations seek to establish partnerships or alliances with key stakeholders, and online communities of social networks represent an optimal tool to achieve it [47]. HOs with fewer fans operate differently from those with a greater number of followers [24], missing the potential of social media to reach a larger audience [1]. HOs that want to enhance the growth of their online community must be willing to develop content and dialogues that encourage interaction [48] since, if they do not, the online community may be weakened [49].

Likewise, the size of the online community encourages the development of social media content [50]. In an environment characterized by a growing consumer distrust towards corporate messages, sharing information through these platforms (when the user understands that this information is beneficial) is a powerful way to increase consumer confidence, thus maintaining and strengthening the online community and maximizing user participation in order to generate word of mouth marketing [24].

Based on the above, the following hypothesis is proposed:

**Hypothesis** **4** **(H4).** *The size of the online community affects the level of users’ online commitment to HOs via social media.*

### 2.5. Format

The type of format through which content can be disseminated on social networks (photo, video, link, etc.) is a key aspect to assessing the quality of the communication [51]. In addition, the different types of formats exhibit different levels of interactivity, expressed through the degree to which users can influence the form and content of the media environment [52]. Multimedia has proven to be the format that has the potential to be engaging for users because of its direct impact on various senses [53]. The photo format, according to [54], is especially appropriate for health communication, due to its impact on people’s knowledge, attitudes and perceptions regarding health issues.

The authors of [24] analyze the relationship between the use of different types of formats and the level of user engagement, concluding that although the video format is the most attractive for users, its presence is scarce in the social networks of the sampled HOs. On the contrary, the link format and the publications made through text only are always present in social networks, but in general, they are not considered attractive [14]. The authors of [25,55] identify that posts with a good engagement rate are significantly associated with a video format, although the most common format in posts are the link and the photo format.

Considering previous literature, the following hypothesis is posed:

**Hypothesis** **5** **(H5).** *The message format affects the level of users’ online commitment to HOs via social media.*

### 2.6. Content

The ability of the message’s content to capture the user’s attention directly influences the scope of the information disclosed [24]. Social media users mainly share information on these platforms when they believe that the information is beneficial to others [56]. Thus, the shared information can contribute to maintaining and strengthening the online community, being an incentive to maximize the user’s commitment to the organization by generating “word of mouth marketing” [24].

Although the content of the message disclosed is a factor that has been recently incorporated into the analysis of health communication through social media, its contribution to the generation of engagement in the context of HOs would require further study. Previous research is heterogeneous with respect to the criteria used to classify message content and which is the most appropriate methodology to analyze the information but agrees in that the most recurrent content does not necessarily generates greater engagement.

In this sense, [57] show that organization promotion was the dominant content type across posts, using the social network as a unidirectional communication channel, rather than encouraging participation and engagement for users. The authors of [14] use the National Library of Medicine’s Medical Text Indexer to perform semantic groups, discovering that posts about ”activities and behaviors” and “phenomenon” are positively associated with the level of engagement, despite the fact that the content “concepts and ideas” is the most recurring among posts. For [11], the categories that generated the greatest engagement were “testimonies”, “solidarity” and “anniversaries”, despite not being the most frequent topics. The authors of [25] state that posts with a good engagement rate were significantly associated with a health education post and a risk communication post. Finally, literature shows that controversial issues such as vaccination, or sensitive topics as cancer, attract the attention of users and generate debate [55,58] and that the audience is willing to commit when the organization provides relevant educational and news content [59].

Thus, the following hypothesis is posited:

**Hypothesis** **6** **(H6).** *The content of posts affects the level of users’ online commitment to HOs via social media.*

## 3. Materials and Methods

### 3.1. Sample

The Spanish health system enjoys a very good reputation internationally, being considered in the “top ten” in the ranking developed by the World Health Organization for 2019, which includes 190 health systems from 190 countries. Therefore, it is considered appropriate to explore the Spanish case as a reference for this study. The initial sample included 163 Spanish public and private hospitals, gathered from the 2018 National Hospitals Catalog published by the Spanish Ministry of Health, Consumption and Social Security. Likewise, among all the social networks used in the field of health communication, Facebook stands out [14] for being the most popular social network worldwide, with more than 2.3 billion active users during 2019 [12]. In fact, HOs acknowledge they use Facebook to engage their target market [24], in addition to their multiple benefits for health communication such as increased social interaction, the availability of information, shared and tailored, the boost of public health surveillance and the potential to influence health policy [11].

The final sample consists of 126 HOs, excluding those 37 lacking a Fan page and/or available statistics provided by Facebook. Data was collected in September 2019 with data from 1st January 2018 to 31st December 2018, in order to show the latest trends in HOs online communication management. Based on previous studies [25], covering 12 months is an acceptable time frame to analyze the information present in social networking.

### 3.2. Methodology

In line with previous studies [14,25], this study assumes that the factors determining the level of online commitment reached by HOs through their fan pages exhibit a linear relationship. This study applies ordinary least squares (OLS) estimation process using IBM SPSS Statistics version 26(International Business Machines, New York, United States). The degree of users’ online is proxied by the online engagement index (E) (Table 2), built from a set of metrics identified in the literature [45]. The index measures the users’ engagement through interactive behaviors: number of likes, comments and shares of a post, which define the three main dimensions of the indicator: popularity (P), commitment (C) and virality (V).

Different software programs are available for social-media data gathering and quantitative analysis; others are designed to analyze the data from a linguistic point of view, specifically regarding content and emotions [62]. Despite the existence of these, the authors and their research group have developed an ad hoc tool (called “Facebook data model”) for this type of research, based on Microsoft technology and previously tested and used in other studies [45,63].

**Figure 1:**
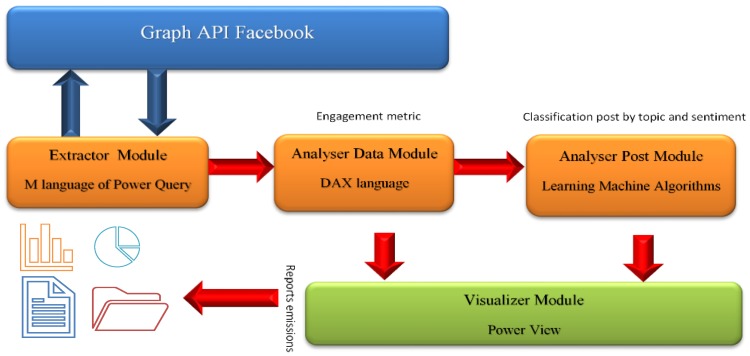
Software Architecture.

This tool (Figure 1) consists of four modules: (1) the extraction module is responsible for retrieving data available from Facebook pages using queries to the Facebook Graph API (application programming interface) based on Power Query M language, (2) the analysis module, developed on Power Business Intelligence (business intelligence tool based on Microsoft’s productivity cloud that allows us to unite different data sources), uses data analysis expressions (DAX language) for developing a star analytic model for the multidimensional exploitation of extracted information, calculating the different items defined in this study, (3) data visualization module uses Microsoft Power View technology to carry out the graphical representation of the results to allow a dynamic analysis of the information (4) and the content analysis module, based on Learning Machine technology, analyses and classifies data into different topics previously parameterized based on a preliminary analysis that helped to define the categories according to the content of the posts of Facebook pages. In addition, sentiment analysis algorithms through “Azure Machine Learning: Analysis” were used to identify sentiments (positive, negative and neutral) in the written posts.

It is important to highlight that this tool was granted permission by Facebook to connect with its Graph API during the time in which this research was developed, although the Cambridge Analytics scandal in early 2018 forced Facebook to cut data access to many applications, including ours. However, after months of waiting for Facebook to grant us access through a formal request (made to its headquarters in the USA), temporary access was granted, which remained active until the end of 2019. Currently, a new process of formal registration of our application has begun, which is being processed by Facebook.

Using the above-detailed software, data collection resulted in 19,817 posts and 32,102 comments (Appendix A). The software provided the data on the file variable, as well as on the following independent variables: online community size, format and content type.

In line with previous studies [45], the size of the online community has been quantified based on the number of fans of each fan page during the period of study. The number of posts for each format is provided directly by the social network and collected through the extraction module. The quantification of the number of posts per topic first required a preliminary analysis to determine the main categories, as well as the parameterization of the analyzer post module and the count of the number of posts within each category with the help of the analysis module.

Regarding the other variables (Table 3), ownership has been measured as a dichotomic variable, taking the value of zero (0) when the property is public and one (1) when private. Economic capacity is measured through GDP per capita corresponding to the province in which the HO operates for 2018 [60]. Finally, among the different alternatives to measure the size of an organization, financial measures such as total assets or net income are commonly used. However, in the health context, the number of beds is a representative measure and often used to quantify the size of HOs [61], thus being considered a control variable [42].

## 4. Results and Discussions

### 4.1. Descriptive Analysis

**Figure 2:**
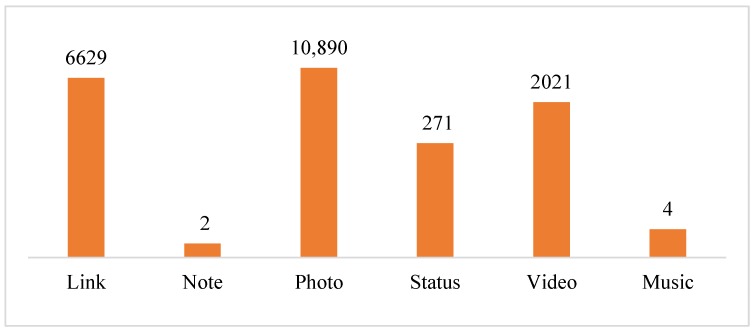
Number of posts by format type.

In order to contextualize the users’ online commitment with the HOs, a descriptive analysis is first performed. Among the formats allowed in Facebook (photo, video, status, link, etc.), HOs mainly use photos 54.95% of the time, in line with previous studies [24,55], while 33.45% of the posts are in link format and only 10.20% of the posts analyzed are videos (Figure 2). These results are in line with [54], who argue that the photo format is especially appropriate for health communication. However, HOs may be missing an opportunity to enhance users’ interaction, since video formats usually attract higher participation rates [25].

**Figure 3:**
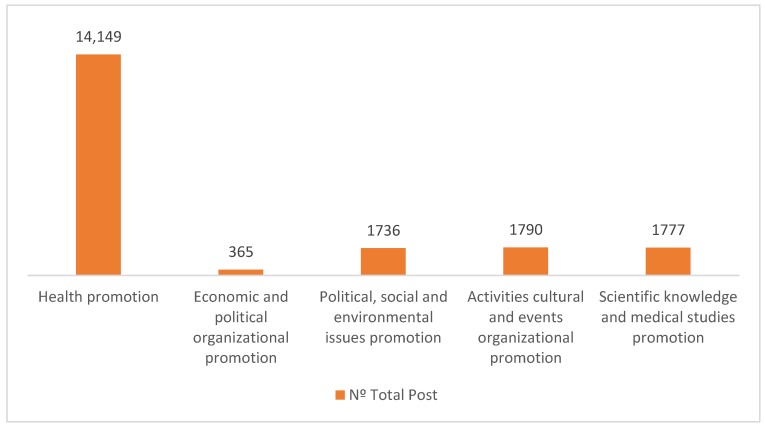
Number of posts by content type.

Regarding content (Figure 3), and as stated by [23], HOs use Facebook mainly to disseminate information on aspects directly related to health promotion (71.40%), such as healthy eating habits or recommendations on sports practices that contribute to the population welfare, advice on self-medication, etc. That is, HOs essentially disseminate content focused on informing and educating the user about aspects related to health improvement. Contrary to the results obtained by [57] for the health industry in the United States and [25] in the case of Malaysia, Spanish HOs do not use Facebook to inform about organizational aspects but for health promotion. In fact, only 9.03% of the posts disclose information related to topics about economic management, current policies or future initiatives that the HO foresees to put into action. Similarly, few posts (8.98%) exhibit the results of medical studies, contributing to disseminate the scientific knowledge developed in fields such as cancer, vaccines and the development of new drugs or scientific discoveries that may affect certain rare diseases, among other issues of marked scientific character. Finally, and to a lesser extent, HOs echo news related to political, social and environmental issues that may affect the health sector (8.76%) on their Facebook pages.

**Figure 4:**
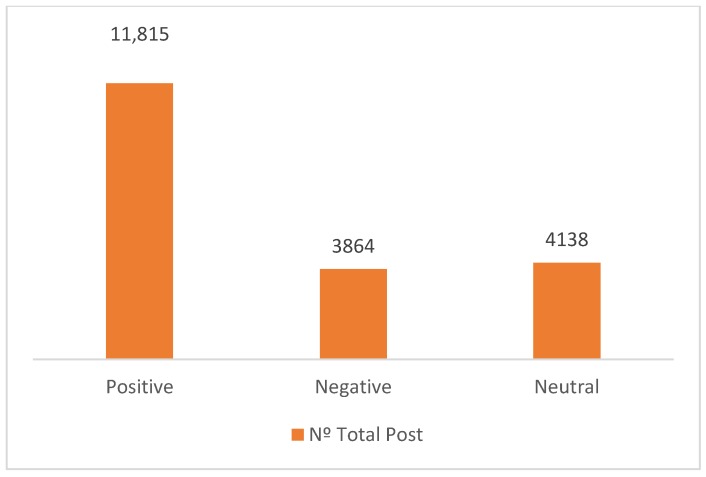
Number of posts by sentiment type.

As [62] point out, the linguistic usage is an interesting characteristic to consider in the context of social media, and many sentiment lexica have been developed to classify post messages, generally as positive and negative. In this sense, [14] state that Facebook posts are generally positive. In this line, our results show that, since 59.62% of the posts show positive expressions, the remaining 40.38% is distributed almost equally between expressions that show negative feelings or are made with expressions of a neutral nature (Figure 4).

**Figure 5:**
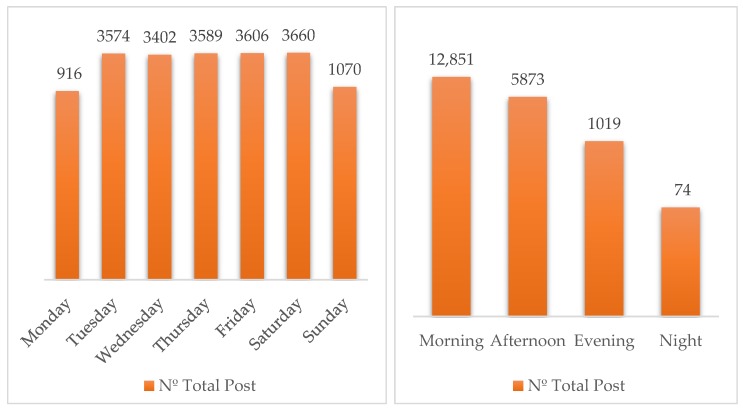
Number of posts per day of the week and per time of the day.

Regarding temporal factors (Figure 5), most posts (64.85%) are published in the morning (6:00 to 12:59), followed by a 29.64% of posts issued in the afternoon (13:00 to 18:59) and evening (19:00 to 24:00, 5.14%), being the night period (00:00 to 05:59) the least frequent posting time (0.37%). The HOs’ behavior is consistent with [55], for whom making a Facebook post during an active period (similar to working hours) will achieve a significantly higher participation rate, but differs from the recommendations given by [25], who advise publishing at night to attain a higher participation rate. 

As for the days of the week, Saturday presents the largest number of posts issued, followed very closely on Friday, with fewer posts on Monday and Sunday. However, in line with previous studies [25], the days accumulating the largest number of publications, representing 76.13% are from Monday to Friday.

Focusing on the main parameters of the engagement index, Table 4 shows that the 19,817 posts analyzed in this study receive 526,495 “likes”, 32,102 comments and 232,977 shares. The average of “likes” per post is 26.56, comments averaging 1.62 and shares 11.75. Given these initial results, quick and simple actions predominate [11].

Table 5 details the average values of the engagement index (E) and the dimensions from which it is built: popularity (P), commitment (C) and virality (V). The average value of E shows a low level of heterogeneity in terms of user engagement (mean 36.76, standard deviation 29.34). The average value of popularity (24.55) is remarkably higher than that of commitment (1.40) and virality (10.80). Unlike the engagement index, the results of the standard deviation of the three dimensions would indicate a greater diversity.

According to the results of engagement (E) and the three individual dimensions, users engage with HOs mainly through “likes”. Popularity is the most popular form of commitment, but this action, which requires a low investment in time and effort, is considered a low-engagement type, because it is the simplest and fastest action among the three options offered by Facebook [11]. An interesting finding is that “shares” are used more widely than “comments”, so it seems that users like to share the relevant content published by HOs, but Virality does not imply a high type of engagement either.

The high levels of popularity show that users like most of the posts and consider that the posts made by the HOs are of interest, but they do not show an additional interest in sharing the information or engaging in a dialogue with the organization by making comments. These results suggest a limited interest on the part of the users to start conversations with the HO, so there exists a missing link between them and the organization.

These results are a sign of the need to continue advancing in the knowledge about health communication in social networks, in order to understand which dynamics HO’s must carry out on Facebook, to help them take advantage of the full potential of this means of communication and engage users with the organization. 

### 4.2. Determinants of the Level of Users’ Engagement via Facebook 

According to the multiple regression analysis (Table 6), the proposed model exhibits an explanatory ability of 53.10%, and linearity is corroborated by Fisher’s F-test. In addition, after confirming the previous hypothesis related to normality, homoscedasticity, independence and collinearity of the aforementioned methodological approach, the Pearson correlation matrix (Appendix B) shows that, although there is a medium-strength correlation between some of the independent variables, multicollinearity do not affect the proposed model [64].

In terms of significance, seven of the proposed variables are statistically significant, supporting their choice as relevant for the model. The variables “Ownership”, “Economic capacity”, “Size of the online community”, “Size of the organization”, “Type of format” and “Type of content” contribute significantly to explain the level of engagement achieved by HOs through Facebook. 

Regarding the hypotheses posited, results support H1; that is, the ownership of the organization affects the level of engagement achieved by HOs. In contrast to previous studies conducted in the Spanish context [1], which evince the best use of social networks by private hospitals and its best engagement metrics, our results confirm that users are more committed to public HOs.

The Spanish public health system is subject to continuous social scrutiny and is one of the basic pillars of the welfare state. This result is reasonable and may be justified by the fact that users perceive greater utility and show greater confidence in the messages disseminated by the public system, understanding that they are aimed at the common good. This leads us to think that public HOs concentrate their efforts on achieving a higher level of social media engagement, which would be a reflection of the effectiveness with which they are carrying out the health communication [21].

According to [37], users who live in more affluent cities are more likely to use social networks. However, our results indicate that, at a regional level, users living in regions with fewer economic resources are more committed to HOs via social media, thus confirming H2. These users have probably a limited health supply, either because they lack the sanitary infrastructure that a large city could provide, because they cannot easily access it (remote from the population center) or because of the increased cost of certain health services [38]. Social media opens a way for users with lower economic capacity to have access to information on health-related issues, which benefit HOs through greater participation and involvement in the organization through the fan page. 

Larger HOs have more resources and, therefore, are more likely to implement social networks in their communication strategy [46]. According to the results presented, those HOs reach higher levels of engagement. Thus, H3 can be confirmed in the sense that the size of the organization is a determining factor of online participation levels through their fan pages. However, size should not represent a barrier to the generation of online engagement, since smaller organizations, and with a smaller budget, have an opportunity to use social networks to connect with their users at a reduced cost [44]. As the results of [45] show, smaller organizations are an example of how organizations with very little capacity have to implement and use new technologies and how social media can be used strategically to involve and engage stakeholders with the organizations goals.

Regarding H4, the results confirm that the size of the online community positively affects the level of online commitment. The HOs seem to be taking advantage of the potential of social media to reach a wide audience [1], which in turn has an impact on the achievement of higher levels of engagement. In this sense, if HOs want to enhance the growth of their online community, they must be willing to develop content and dialogues that foster interaction [48] in order to maximize user participation, as well as to promote word-of-mouth marketing [24].

Likewise, posting through a specific format significantly influences engagement levels, as expected in H5. Specifically, the video format generates greater user participation, while making publications in other less interactive formats (link, note, music or status) has the opposite effect. In the previous literature, there seems to be some consensus regarding the popularity of the photo format [24,55], although some studies have found that less “interactive” posts in text or link format were more commonly used on Facebook pages than media-rich posts [14,65]. The results signal that publishing content through enriched media leads to a higher level of engagement. Specifically, the video format has a significant effect, perhaps for being especially appropriate for health communication [54], although the photo format is the most recurrent [24,25].

Finally, the findings regarding the variable “type of content” confirm H6, albeit with some nuances. Of the five topic categories under which the contents of the posts were classified, only one, related to the dissemination of scientific knowledge developed in fields such as cancer, vaccines, new drugs or results of different medical studies, positively influences the level of online engagement with HOs. In this sense, the HOs analyzed take advantage of the interactive potential of controversial health topics to generate debate [55,58], in order to attract the user and trigger a reaction which leads them to engage with the organization. Another interesting finding is that posts related to health promotion have a greater specific weight in the classification of topics [23]. However, they do not have a significant effect on the users’ commitment, contradicting the results of [25], who associate the highest participation rates with health educational posts. This could be due to the fact that this is the content that users expect to find in HOs’ social networks, not finding it relevant and are therefore not motivated to engage in further interaction beyond the initial access to the information. Therefore, HOs must take special care in the content disclosed, in that it must be relevant to the user [59], and should move away from informative content to promote the organization, since users can perceive that this type of posts focus more on the interest of the organization than on the public interest [14].

## 5. Conclusions

Social networks provide health communication with multiple benefits for users/patients and health organizations alike. Social media allow HOs to create an innovative and bidirectional communication channel between doctors, patients and the researchers, through which users’ increasing demand for information and participation on health-related issues can be met. From an organizational standpoint, embedding social media into the HO´s communication strategies can help these organizations to increase their visibility in the market, strengthen their corporate image and strategic position and reach their target audience. Therefore, and in line with previous literature, improving the effectiveness of health communication should involve encouraging user participation in HOs’ social networks. Thus, the main aim of this study is to identify the main factors that could influence the level of online users’ commitment.

The relevance of the health-related information contrasts with the (sometimes) difficult understanding of its implications. Our findings show that HOs could improve their users’ engagement by encouraging them to express their feelings, opinions or concerns through “comments” and posting information through attractive, visually rich and interactive formats (such as videos). Furthermore, users show great interest in content related to scientific knowledge, especially regarding controversial topics (such as vaccinations or new drugs) or topics in which there is a particular social awareness (i.e., cancer), urgency or lack of knowledge (such as the Ebola epidemic in 2014–2016 or the recent COVID-19 pandemic thread in 2020). HOs may also be missing opportunities to connect with their audience, since the hours and days of posting are not optimally exploited. HOs should reschedule both the posting day and the time of the day beyond business days or hours. In this regard, technological advances could help in this task, programming the content publication at a time that is more likely to generate user interaction, such as evening hours and during the weekend. This combined strategy could allow HOs to reach a wider audience and to achieve higher levels of engagement, disseminating health related information in a simple but effective way, and meeting the user’s needs for knowledge and the establishment of bidirectional communication with health experts.

Although the topic, format and timing variables behave similarly throughout the whole sample, some organizational differences arise. The results show that public entities are more efficient in managing their online communication strategy than their private counterparts and that the largest HOs exhibit better results in terms of engagement. These results point out that public and larger HOs are much more aware of the resources they have at their disposal to disseminate information and engage their users, generating value without increasing their costs. Furthermore, users living in poorer regions are more involved with HOs, so it is possible that they may be missing an opportunity by not connecting with large audiences in more affluent regions, which are critically important for private HOs.

Our findings contribute to the existing theories mentioned in the literature review. According to the Dialogic Communication Theory, social media are a valuable channel for health communication, especially in less favored areas in view of the greater commitment they exhibit with HOs. Regarding Resource Dependence Theory, smaller HOs should take advantage of the interactive potential offered by social media to reduce uncertainty and resource dependence, due to the optimal relationship between assuming lower costs of implementation and maintenance and reaching a wider audience. Pertaining to Media Richness Theory, our findings support the view that not all formats present the same level of engagement, with video and photos having a greater impact on users’ engagement. Finally, it is also worth noting the relevance of the Uses and Gratifications Theory, since the findings of our research confirm that, in order to be effective, HOs should ensure that posts are relevant and in line with users’ interests. This is particularly true in the case of information about scientific knowledge and medical studies which have shown to enhance users’ commitment to an HO.

This study expands the existing literature on health communication through social media, with the added value of focusing on the HOs belonging to one of the most reputable health systems worldwide, the Spanish health system. Therefore, knowing what is being done at the level of dissemination of health information, this may be relevant for other areas to improve the commitment of users with the organization, constituting a benchmark for environments where the health system suffers from low levels of commitment. For those involved in the management of HOs, the benefits offered by social media to improve relations with their users are highlighted, and concrete actions and recommendations that HOs can follow at the organizational level and with respect to their communication policy are included.

Despite its findings, this work has some limitations, such as the selection of hospital, clinic and healthcare organizations and the limited study period of one year. Furthermore, the treatment of photo-based posts has represented an added difficulty, and data downloaded with the Facebook API depends on the organization’s privacy settings and some explicative factors that have not been considered. These limitations could be overcome in future research and lead to new studies. It would be interesting to carry out research in areas where the health system is not as well considered, as in the case of underdeveloped countries, in order to establish a comparison that would help to address possible deficiencies in health communication in the latter. In addition, new research delimiting the medical specialty of the organization and the organization´s reputation, as well as analyzing other actors involved in health communication, would help to understand the social media phenomenon. Similarly, other factors in addition to those considered in this study would be interesting to explore, such as the sentiment of messages or a specific analysis of the audiovisual content posted by the organization. Likewise, with the aim of contributing to an expansion and enrichment of the current literature on this topic, a more in-depth content analysis could be performed by applying different emotion lexicon or by establishing a classification criterion of topics based on the preferences of Facebook users identified through a previous survey.

## Figures and Tables

**Table 1:** Working hypotheses. HO: health organizations.

Hypothesis Number	Hypothesis	Independent Variables
**H1**	The HO´s ownership affects the level of users’ online commitment with HOs via social media.	Ownership
**H2**	The economic capacity of the population affects the level of users’ online commitment with HOs via social media.	Economic capacity
**H3**	The size of the organization affects the level of users’ online commitment with HOs via social media.	Size
**H4**	The size of the online community affects the level of users’ online commitment with HOs via social media.	Online community size
**H5**	The message format affects the level of users’ online commitment with HOs via social media.	Format

**Table 2:** Metrics used to measure user online engagement.

Name	Sign	Formula	Measures
**Popularity**	P	Total likes/total posts	Average number of likes per post
**Commitment**	C	Total comments/total posts	Average number of users’ comments per post
**Virality**	V	Total shares/total posts	Average number of shares per post
**Engagement**	E	P + C + V	Index of online engagement

Popularity (P), commitment (C), virality (V) and the online engagement index (E).

**Table 3:** Independent variables.

Hypothesis	Independent and Control Variables	Measurement
**H1**	**Ownership**	0, public ownership 1, private ownership
**H2**	**Economic capacity**	Regional GDP per capita [60]
**H3**	**Size**	Total number of beds available in the HO [61]
**H4**	**Online community size**	Number of fans of a fan page during the analyzed period.
**H5**	**Format**	
	Photo format	Total number of posts with photos.
	Video format	Total number of posts with videos.
	Other formats	Total number of posts with link, music, note and status.
**H6**	**Content**	
	Health promotion	Natural logarithm of the number of posts with a given topic type.
	Economic and political organizational promotion	
	Political, social and environmental issues promotion	
	Activities cultural and events organizational promotion	
	Scientific knowledge and medical studies promotion	

**Table 4:** Posts’ quantitative parameters.

	Posts	Likes	Comments	Shares
**Total**	19,817	526,495	32,102	232,977
**Mean**	157.277	4178.532	254.778	1849.024
**Standard Deviation**	113.967	4701.223	577.586	2289.250

**Table 5:** Descriptive statistics of the engagement index, popularity, commitment and virality.

	Engagement (E)	Popularity (P)	Commitment (C)	Virality (V)
**Mean**	36.765	24.559	1.400	10.806
**Maximum**	126.613	87.636	30.820	46.373
**Minimum**	1.091	0.599	19.753	0.000
**Standard Deviation**	29.342	19.067	2.125	10.179

Note. N = 126.

**Table 6:** Results of the Regression Analysis.

	Unstandardized Coefficients	Std. Error	t	Sig.
(Constant)	30.182	13..276	2.273	0.025
Ownership	−8.784	4.982	−1.763	0.081 *
Economic capacity	−0.708	0.387	−1.831	0.070 *
Size of the organization	0.045	0.010	4.644	0.000 ***
Online community size	0.004	0.001	5.902	0.000 ***
Photo format	−0.074	0.047	−1.568	0.120
Video format	0.189	0.110	1.727	0.087 *
Other formats	−0.151	0.046	−3.268	0.001 ***
Health promotion	1.023	2.945	0.347	0.729
Economic and political organizational promotion	−0.895	3.053	−0.293	0.770
Political, social, and environmental issues promotion	−1.953	2.549	−0.766	0.445
Activities cultural and events organizational promotion	−0.143	2.487	−0.057	0.954
Scientific knowledge and medical studies promotion	8.370	3.331	2.513	0.013 **
R−squared			0.531	
Durbin−Watson			2.176	
F−Fisher test			10.668 (0.00 ***)	

Significant at: *p* < 0.01 ***; *p* < 0.05 **; *p* < 0.1 *.

## Appendix A

**Health Organization**	**Nº Post**	**Nº Comments**	**Nº Likes**	**Nº Shares**
HO1	14	2	220	63
HO2	187	2	112	90
HO3	275	956	15,927	8736
HO4	507	30	2942	1465
HO5	141	191	5109	1855
HO6	151	893	10,189	4401
HO7	41	119	1752	613
HO8	229	204	5867	2348
HO9	25	23	822	447
HO10	53	17	851	221
HO11	117	171	3,213	1519
HO12	55	5	774	195
HO13	331	721	13,859	4778
HO14	352	804	16,504	9887
HO15	48	137	1121	385
HO16	203	504	8953	3456
HO17	187	57	2518	1044
HO18	127	262	3370	1924
HO19	161	6	283	120
HO20	92	15	632	41
HO21	165	27	1257	260
HO22	304	295	7983	2,329
HO23	202	79	2601	604
HO24	180	25	1214	397
HO25	192	77	2051	347
HO26	3	0	3	1
HO27	49	15	280	77
HO28	38	143	1981	1159
HO29	89	33	1621	571
HO30	40	2	129	16
HO31	57	1	233	229
HO32	383	211	8281	4520
HO33	188	105	8000	2498
HO34	81	34	1518	593
HO35	5	4	277	45
HO36	186	738	14,305	7153
HO37	181	445	6393	1026
HO38	323	218	11,445	5182
HO39	279	412	18,413	6126
HO40	4	1	19	10
HO41	2	1	38	5
HO42	42	10	438	11
HO43	1	0	4	0
HO44	107	40	976	387
HO45	93	560	2250	1181
HO46	186	509	7035	5260
HO47	72	188	2815	1517
HO48	18	14	194	106
HO49	193	190	3668	1249
HO50	223	167	9449	1611
HO51	41	77	1138	348
HO52	122	425	4653	2578
HO53	186	284	5205	5818
HO54	481	914	13,028	4762
HO55	97	12	1462	429
HO56	104	194	2433	759
HO57	203	715	10,437	9194
HO58	66	72	649	1380
HO59	335	836	9192	7459
HO60	11	3	52	12
HO61	258	324	6318	2270
HO62	236	53	1726	371
HO63	222	161	4169	2168
HO64	34	15	1069	264
HO65	36	2	75	9
HO66	28	35	995	355
HO67	133	60	1487	418
HO68	60	12	374	94
HO69	256	183	4774	688
HO70	27	14	300	158
HO71	225	48	2056	1015
HO72	293	808	12,649	4053
HO73	149	7	257	99
HO74	207	75	3343	1278
HO75	105	62	1427	311
HO76	283	109	5018	2239
HO77	73	15	791	271
HO78	213	111	6027	1810
HO79	294	348	9474	2487
HO80	254	47	2652	649
HO81	178	56	2180	856
HO82	288	183	6167	2997
HO83	294	243	3591	1014
HO84	327	71	2533	1092
HO85	315	40	1834	658
HO86	103	293	3710	1069
HO87	111	102	1559	236
HO88	179	34	1209	505
HO89	37	129	2661	1201
HO90	367	333	11,214	5083
HO91	15	32	819	339
HO92	287	285	9457	3196
HO93	92	19	302	178
HO94	279	1689	11,448	7799
HO95	90	45	1277	485
HO96	150	208	4394	4018
HO97	108	96	1702	467
HO98	84	63	1624	1317
HO99	287	404	5280	2928
HO100	56	217	2407	967
HO101	495	296	4744	1353
HO102	5	0	31	31
HO103	54	30	601	507
HO104	198	229	1943	944
HO105	76	194	2586	1809
HO106	67	40	1351	313
HO107	19	0	63	37
HO108	295	5827	9762	3936
HO109	176	27	903	89
HO110	225	1090	19,718	7680
HO111	108	96	1783	767
HO112	73	216	4433	2274
HO113	358	497	12,401	5444
HO114	80	168	2590	2012
HO115	119	107	1516	1047
HO116	289	926	16,452	5774
HO117	210	85	2329	2296
HO118	109	155	2706	1481
HO119	44	12	652	250
HO120	242	1047	18,728	6420
HO121	100	190	5557	1217
HO122	46	27	336	110
HO123	100	2	161	11
HO124	158	345	10,945	7327
HO125	74	81	1334	518
HO126	236	189	4383	1121
Total	19,817	32,102	526,495	232,977
Mean per page	157.278	254.778	4178.532	1849.024

## Appendix B

		**Engagement**	**Ownership**	**Economic Capacity**	**Size**	**Online Community Size**	**Photo Format**	**Video Format**	**Other Formats**	**Health Promotion**	**Economic and Political Organizational Promotion**	**Political, Social and Environmental Issues Promotion**	**Activities Cultural and Events Organizational Promotion**	**Scientific Knowledge and Medical Studies Promotion**
**Pearson correlation matrix**	Engagement	1.000	−0.361	−0.174	0.484	0.424	0.190	0.251	−0.021	0.171	0.162	0.141	0.168	0.325
	Ownership	−0.361	1.000	0.174	−0.433	0.054	−0.055	−0.035	0.095	−0.076	0.022	−0.122	0.002	−0.176
	Economic capacity	−0.174	0.174	1.000	0.021	−0.051	−0.026	0.049	0.093	0.005	0.067	0.085	0.017	0.039
	Size	0.484	−0.433	0.021	1.000	0.082	0.160	0.061	−0.012	0.113	0.119	0.299	0.062	0.234
	Online community size	0.424	0.054	−0.051	0.082	1.000	0.325	0.274	0.392	0.312	0.321	0.240	0.287	0.348
	Photo format	0.190	−0.055	−0.026	0.160	0.325	10.000	0.337	0.266	0.666	0.577	0.552	0.570	0.693
	Video format	0.251	−0.035	0.049	0.061	0.274	0.337	10.000	0.319	0.481	0.182	0.173	0.374	0.423
	Other formats	−0.021	0.095	0.093	−0.012	0.392	0.266	0.319	1.000	0.609	0.261	0.388	0.407	0.502
	Health promotion	0.171	−0.076	0.005	0.113	0.312	0.666	0.481	0.609	1.000	0.386	0.484	0.599	0.724
	Economic and political organizational promotion	0.162	0.022	0.067	0.119	0.321	0.577	0.182	0.261	0.386	1.000	0.403	0.564	0.592
	Political, social and environmental issues promotion	0.141	−0.122	0.085	0.299	0.240	0.552	0.173	0.388	0.484	0.403	1.000	0.469	0.560
	Activities cultural and events organizational promotion	0.168	0.002	0.017	0.062	0.287	0.570	0.374	0.407	0.599	0.564	0.469	1.000	0.708
	Scientific knowledge and medical studies promotion	0.325	−0.176	0.039	0.234	0.348	0.693	0.423	0.502	0.724	0.592	0.560	0.708	1.000
**Sig. (unilateral)**	Engagement	−	0.000	0.025	0.000	0.000	0.017	0.002	0.408	0.028	0.035	0.058	0.030	0.000
	Ownership	0.000	−	0.026	0.000	0.275	0.269	0.347	0.144	0.200	0.402	0.087	0.493	0.024
	Economic capacity	0.025	0.026	−	0.407	0.286	0.388	0.292	0.151	0.477	0.228	0.172	0.424	0.334
	Size	0.000	0.000	0.407	−	0.180	0.037	0.247	0.448	0.103	0.092	0.000	0.245	0.004
	Online community size	0.000	0.275	0.286	0.180	−	0.000	0.001	0.000	0.000	0.000	0.003	0.001	0.000
	Photo format	0.017	0.269	0.388	0.037	0.000	−	0.000	0.001	0.000	0.000	0.000	0.000	0.000
	Video format	0.002	0.347	0.292	0.247	0.001	0.000	−	0.000	0.000	0.020	0.026	0.000	0.000
	Other formats	0.408	0.144	0.151	0.448	0.000	0.001	0.000	−	0.000	0.002	0.000	0.000	0.000
	Health promotion	0.028	0.200	0.477	0.103	0.000	0.000	0.000	0.000	−	0.000	0.000	0.000	0.000
	Economic and political organizational promotion	0.035	0.402	0.228	0.092	0.000	0.000	0.020	0.002	0.000	−	0.000	0.000	0.000
	Political, social and environmental issues promotion	0.058	0.087	0.172	0.000	0.003	0.000	0.026	0.000	0.000	0.000	−	0.000	0.000
	Activities cultural and events organizational promotion	0.030	0.493	0.424	0.245	0.001	0.000	0.000	0.000	0.000	0.000	0.000	−	0.000
	Scientific knowledge and medical studies promotion	0.000	0.024	0.334	0.004	0.000	0.000	0.000	0.000	0.000	0.000	0.000	0.000	−
